# Comparison of Letrozole Versus Clomiphene Citrate Combined With Human Menopausal Gonadotropin in Women With Clomiphene-Resistant Anovulatory Infertility Undergoing Intrauterine Insemination: A Retrospective Cohort Study

**DOI:** 10.7759/cureus.88802

**Published:** 2025-07-26

**Authors:** Tithi Barman, Vinita Singh, Nilajkumar D Bagde

**Affiliations:** 1 Obstetrics and Gynaecology, All India Institute of Medical Sciences, Raipur, IND

**Keywords:** clomiphene citrate, human menopausal gonadotropin, infertility, intrauterine insemination, letrozole, ovulation induction

## Abstract

Background

Millions of people of reproductive age worldwide struggle with infertility. Treatments such as controlled ovarian hyperstimulation and intrauterine insemination (IUI) are commonly used to address various causes of infertility. Medications such as clomiphene citrate (CC) and letrozole, when combined with human menopausal gonadotropin (HMG), have been shown to improve pregnancy rates and reduce treatment costs.

Objective

This study aimed to compare the effect of minimal stimulation using HMG with either CC or letrozole for ovulation induction in women with CC-resistant anovulatory infertility. It also assessed the impact of these combinations on follicular development and endometrial thickness.

Materials and methods

This retrospective cohort study included data from 84 infertile women diagnosed with polycystic ovary syndrome (PCOS). Participant records were retrieved from the hospital database at AIIMS, Raipur, India, covering the period from April 2021 to August 2022. Women were divided into two groups based on their treatment: one group received CC 100 mg and the other letrozole 5 mg, both in combination with HMG administered on alternate days. Forty-two women received letrozole + HMG and 40 received CC + HMG. Two participants in the CC group were excluded due to the development of four dominant follicles. Transvaginal ultrasound was performed on alternate days until follicles exceeded 17 mm. IUI was carried out 36 hours after HCG administration. Fourteen days later, a urinary pregnancy test was done if the participant had not yet menstruated. The clinical pregnancy rates were compared between the two groups.

Results

There was no significant difference between the two groups in terms of mean follicular diameter, number of dominant follicles, endometrial thickness, HMG dosage required, or the number of days to achieve dominant follicle formation. The IUI outcomes were also comparable in both groups.

Conclusion

The study concludes that both letrozole and CC, when combined with HMGs, are equally effective in inducing ovulation and achieving successful IUI outcomes in women with CC-resistant anovulatory infertility.

## Introduction

Infertility affects millions of individuals of reproductive age globally. According to current estimates, one in six people will experience infertility at some point in their lives. It is defined as the inability to conceive after at least one year of regular, unprotected sexual intercourse [1].

Infertility not only affects physical health but also significantly impacts quality of life. It can lead to psychological distress, social stigma, financial burdens, and marital conflict [2].

Approximately 186 million people worldwide are believed to be affected. Primary infertility refers to the inability to achieve pregnancy, while secondary infertility describes difficulty in conceiving again after a previous pregnancy. Globally, up to 15% of reproductive-age couples struggle with infertility, and in India, the World Health Organization reports that 3.9% to 16.8% of women are affected by primary infertility [3].

Controlled ovarian hyperstimulation combined with intrauterine insemination (IUI) is commonly used to treat a range of infertility causes, including unexplained infertility, anovulation, early-stage endometriosis, and borderline male factor infertility [4].

Anovulation is responsible for about 30% of female infertility cases, with polycystic ovarian syndrome (PCOS) being a primary cause [5].

Two key oral medications used for ovulation induction are clomiphene citrate (CC), a selective estrogen receptor modulator, and letrozole, a reversible non-steroidal aromatase inhibitor.

Letrozole works by blocking the conversion of androgens to estrogens, thereby stimulating the release of follicle-stimulating hormone (FSH). It is often associated with mono-ovulation and has minimal negative effects on the endometrium or cervical mucus, making it a favorable alternative to CC in anovulatory infertility [6].

CC acts on the hypothalamic-pituitary-gonadal axis by blocking estrogen receptors (ERs) in the hypothalamus, which disrupts the negative feedback mechanism and increases FSH production [6]. CC can induce ovulation in 60%-85% of anovulatory women, though conception rates remain lower, around 10%-20% per cycle. This discrepancy is attributed to CC's anti-estrogenic effects, which can lead to long-term depletion of estrogen receptors, adversely affecting endometrial receptivity and cervical mucus [7].

IUI is one of the simplest and most cost-effective assisted reproductive techniques. It is frequently used in cases of mild male factor infertility and unexplained infertility. When combined with ovulation induction, IUI significantly improves pregnancy outcomes [8].

Both CC and letrozole are widely used as first-line agents for ovulation induction in IUI cycles. However, few studies have directly compared their effectiveness when combined with human menopausal gonadotropin (HMG).

Combining CC or letrozole with HMG may reduce the required gonadotropin dose, lower treatment costs, and decrease the risk of ovarian hyperstimulation syndrome (OHSS). However, due to CC’s anti-estrogenic effects on the endometrium, cervical mucus, and uterine blood flow, the pregnancy outcomes may differ between the two combinations [6].

CC can induce ovulation in 60%-85% of women, but only 10%-20% achieve pregnancy per cycle. Additionally, 20%-25% of women develop resistance to CC and fail to ovulate [9]. When CC is combined with HMG, the pregnancy rate may rise to approximately 46% [10]. Letrozole, on the other hand, induces ovulation in 52.3% of cases, with a pregnancy rate of 29.3% when used alone. However, the combination of letrozole and HMG has been reported to raise pregnancy rates to 54.7% [11]. Given these findings, the current study aims to evaluate and compare the effectiveness of CC plus HMG versus letrozole plus HMG in improving IUI outcomes in women with CC-resistant anovulatory infertility.

Minimal stimulation protocols are increasingly considered ideal in ovulation induction, as they help maximize pregnancy rates while reducing complications and costs. They require fewer gonadotropins and shorter stimulation durations, making them more cost-effective compared to conventional gonadotropin-only cycles [12].

The primary objective of this study was to compare the outcomes of minimal stimulation using HMG with CC versus letrozole in inducing ovulation among women with CC-resistant anovulatory infertility. The secondary objectives included evaluation of leading follicle size, number of dominant follicles, endometrial thickness on the day of hCG administration, incidence of multiple pregnancies, luteinized unruptured follicles, functional cyst development, and occurrence of OHSS.

## Materials and methods

Study design

This retrospective cohort study included data from 84 infertile women with PCOS who were referred to the Infertility Outpatient Department at AIIMS, Raipur, India, between April 2021 and August 2022. The study was approved by the Institutional Ethics Committee. Participants were divided into two groups: Group A (letrozole + HMG group) comprising 42 participants who received letrozole 5 mg daily from day 3 to day 7 of the menstrual cycle, combined with HMG 75-150 IU administered on days 5, 7, and 9; and Group B (CC + HMG Group) comprising 40 participants who received CC 100 mg daily from day 3 to day 7, along with the same HMG regimen (75-150 IU on days 5, 7, and 9). Two participants were excluded from this group due to the development of four dominant follicles.

Eligibility criteria

(i) Infertile women aged 21-39 years with PCOS diagnosed as having CC-resistant anovulatory infertility and planned for IUI following ovulation induction, (ii) those with corrected thyroid and prolactin levels, (iii) women whose husband's semen analysis shows a total sperm count > 10 million/mL, and (iv) couples who provided voluntary informed consent were included in the study.

Women (i) not ideal for IUI, such as those with pelvic pathologies that may affect outcomes, including advanced-stage (III or IV) endometriosis, severe adenomyosis, fibroids, endometrial polyps, severe pelvic adhesions, uterine anomalies, or a history of tuberculosis; (ii) with a history of OHSS; (iii) with bilateral tubal pathology; (iv) with a history of adverse reactions to any of the study medications; and (v) whose day 2 antral follicle count (AFC) > 15 were excluded.

Two participants in the CC + HMG group were excluded due to the development of four dominant follicles.

Case definition

Clomiphene resistance is the failure to ovulate after receiving 150 mg of CC daily for six days across at least three cycles, while clomiphene failure is the inability to conceive despite ovulation after taking 150 mg of CC.

Sample size

Based on a previously published study, pregnancy rates confirmed via fetal heart activity by ultrasound were 12.64% for CC + HMG and 26.51% for letrozole + HMG [13].

Sample size was estimated using the following formula: 

$N = \frac{{{(Z_{&alpha;/2}\sqrt{2p(1-p)} + Z_{1-&beta;}\sqrt{p_{1}(1-p_{1})p_{2}(1-p_{2})}})^{2}}}{(p_{1}-p_{2})^{2}}$

where:

$p1 = 12.64$% (pregnancy rate of the CC + HMG group)

$p2 = 26.51$% (pregnancy rate of the letrozole + HMG group)

$p = (0.1264 + 0.2651) / 2 = 0.196)$ (average proportion)

$Z&alpha;/2 = 1.96$ (at 95% CI)

$Z1-&beta; = 0.84$ (at 80% power)

The calculated sample size was 42 participants per group, totaling 84 participants for the study.

The total duration of the study was one year.

Statistical analysis

Data were compiled using Microsoft Excel (Microsoft Corp., Redmond, WA, USA) and analyzed with IBM SPSS Statistics for Windows, Version 26.0 (Released 2018; IBM Corp., Armonk, NY, USA).

For continuous variables not normally distributed, the Mann-Whitney U test was used.

For normally distributed continuous variables, the unpaired t-test was applied.

For categorical variables, Fisher’s exact test and chi-square test were used as appropriate.

## Results

The present study was conducted at the Department of Obstetrics and Gynaecology, AIIMS, Raipur, from April 2021 to August 2022. A total of 84 infertile women between 21 and 39 years of age with CC-resistant anovulatory infertility were enrolled and divided into two groups based on the treatment received: CC + HMG or letrozole + HMG.

Initially, 84 participants were recruited; however, two participants in the CC + HMG group developed four dominant follicles and were therefore excluded from the study. No other adverse effects related to either medication were observed.

The CC + HMG group had a group size of n = 40, while the letrozole + HMG group had n = 42.

Table 1 presents the baseline demographic characteristics of participants in both groups. No statistically significant differences were observed between the two groups in terms of patient age, husband's age, past medical or surgical history, menstrual cycle regularity, duration of infertility, type of infertility (primary or secondary), or presence of male factor infertility.

**Table 1:** Demographic features of Group A (letrozole + HMG) and Group B (CC + HMG) Data are presented as mean ± SD.  For patient's age and duration of infertility, the Mann-Whitney U test was used. For the type of infertility, the chi-square test was used. CC: clomiphene citrate, HMG: human menopausal gonadotropin.

Variables		Group A (n = 42)	Group B (n = 42)	Test value	p-value
Patient's age (years)		29.26 ± 3.49	29.11 ± 4.67	810.5	0.53
Duration of infertility (years)		6.48 ± 2.90	6.92 ± 2.83	810.5	0.519
Type of infertility	Primary	32 (76.2)	31 (73.8)	0.06	0.8
	Secondary	10 (23.8)	11 (26.2)		

There was no statistically significant difference between the two groups regarding key demographic and clinical variables. Specifically, maternal age did not differ significantly (Mann-Whitney U = 810.5, p = 0.52); 

husband's age also showed no significant difference (U = 865, p = 0.87); age group distribution was similar between the groups (χ² = 1.34, p = 0.51); menstrual cycle regularity was identical in both groups (χ² = 0, p = 1.00); duration of infertility did not significantly vary (U = 810.5, p = 0.519); and the type of infertility showed no significant difference (χ² = 0.06, p = 0.80). Among participants, 32 (76.2%) in the letrozole + HMG group and 31 (73.8%) in the CC + HMG group had primary infertility, while 10 (23.8%) in the letrozole + HMG group and 11 (26.2%) in the CC + HMG group had secondary infertility.

Table 2 presents the comparison of follicular development and endometrial thickness between the two groups. Follicular development was assessed based on the number of days required to form a dominant follicle, mean follicular diameter (mm), number of dominant follicles, and total HMG dose required in each group.

**Table 2:** Comparison of follicular development and endometrial thickness in both groups *Mann-Whitney test was used for all the determinants; p = 0.05 is considered statistically significant. HMG: human menopausal gonadotropin.

Parameters	Group A (n = 42)	Group B (n = 40)	Test value	p-value
Dose of HMG (mg)	257.14 ± 41.05	249.3 ± 54.7	702.5	0.116
Days required to form a dominant follicle	14 ± 0.92	14.3 ± 0.76	686	0.126
Mean follicular diameter (mm)	18.3 ± 0.68	18.2 ± 1.18	859.5	0.838
Number of dominant follicles	1.2 ± 0.47	1.2 ± 0.70	5.30	0.835
Endometrial thickness (mm)	7.3 ± 1.2	6.9 ± 1.1	696	0.09

In this study, there was no statistically significant difference between the two groups regarding mean follicular diameter, number of dominant follicles, total HMG dose required, endometrial thickness, or the number of days needed to develop a dominant follicle.

The mean (±SD) HMG dose in the letrozole + HMG group was 257.14 ± 41.05 IU, while in the CC + HMG group, it was 249.3 ± 54.7 IU. This difference was not statistically significant (U = 702.5, p = 0.116).

The mean (±SD) number of days required to develop a dominant follicle was 14.0 ± 0.92 days in the letrozole + HMG group and 14.3 ± 0.76 days in the CC + HMG group, showing no significant difference (U = 686, p = 0.126).

The mean (±SD) follicular diameter was 18.3 ± 0.68 mm in the letrozole + HMG group and 18.2 ± 1.18 mm in the CC + HMG group (U = 859.5, p = 0.838).

Regarding the number of dominant follicles, in the letrozole + HMG group, 34 participants (80.9%) developed one dominant follicle, 7 (16.7%) developed two, and 3 (7.1%) developed three follicles. In the CC + HMG group, 35 participants (83.3%) developed one dominant follicle and 5 (11.9%) developed two. Two participants (4.8%) in the CC + HMG group developed four dominant follicles; thus, HCG was withheld, and they were excluded from IUI. There was no statistically significant difference in the number of dominant follicles between the groups (χ² = 5.3, p = 0.15).

The mean (±SD) endometrial thickness on the day of HCG trigger was 7.3 ± 1.2 mm in the Letrozole + HMG group and 6.9 ± 1.1 mm in the CC + HMG group. This difference was not statistically significant (U = 696, p = 0.09).

Two women in the CC + HMG group developed four dominant follicles; therefore, IUI was not performed for these participants.

Table 3 presents the comparison of IUI outcomes between the two groups. There was no statistically significant difference observed (χ² = 1.01, p = 0.16). In the letrozole + HMG group, 10 women (23.8%) had a positive urinary pregnancy test (UPT), while 32 (76.2%) had a negative result. In comparison, 6 women (15%) in the CC + HMG group were UPT positive, and 34 (85%) were negative.

**Table 3:** Distribution of women according to IUI outcomes *Chi-square test; p-value < 0.05 is considered statistically significant. IUI: intrauterine insemination.

	Group			Chi-square test^*^	
IUI outcome	Group A (%)	Group B (%)	Total N (%)	Test value	p-value
Positive	10 (23.8)	6 (15)	16 (19.5)	1.01	0.16
Negative	32 (76.2)	34 (85)	66 (80.4)		
Total	42 (100)	40 (100)	82 (100)		

**Figure 1:**
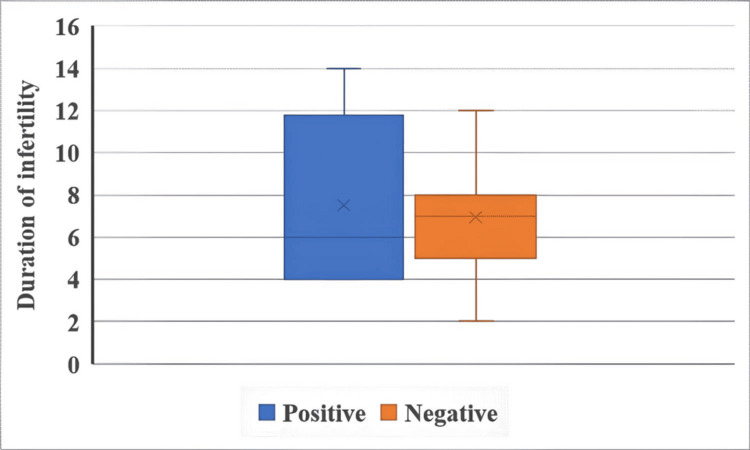
Duration of infertility and IUI outcomes in the letrozole + HMG group The Mann-Whitney U test was used. The duration of infertility was measured in years. p = 0.056. IUI: intrauterine insemination, HMG: human menopausal gonadotropin.

**Figure 2:**
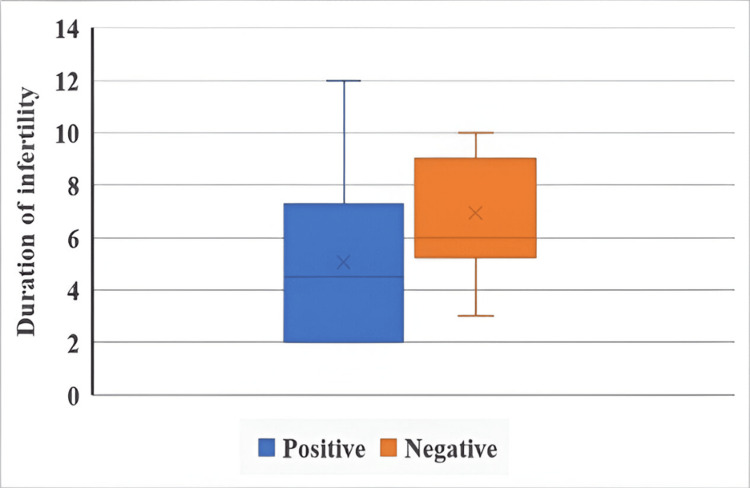
Duration of infertility and IUI outcomes in the CC + HMG group The Mann-Whitney U test was used. The duration of infertility was measured in years. p = 0.631. IUI: intrauterine insemination, CC: clomiphene citrate, HMG: human menopausal gonadotropin.

The duration of infertility and IUI outcomes in both groups are illustrated in Figure 1 and Figure 2.

The duration of infertility showed a borderline significant association with IUI outcome in the letrozole + HMG group (U = 95.5, p = 0.056), suggesting a potential influence on treatment success. However, no such association was observed in the CC + HMG group (U = 143.5, p = 0.631).

**Figure 3:**
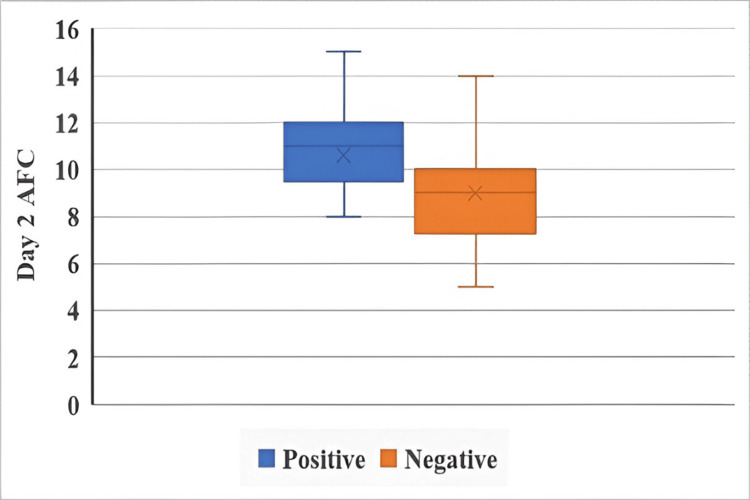
Day 2 AFC and IUI outcomes in the letrozole + HMG group The Mann-Whitney U test was used. p < 0.05 is considered statistically significant. AFC: antral follicle count, IUI: intrauterine insemination, HMG: human menopausal gonadotropin.

**Figure 4:**
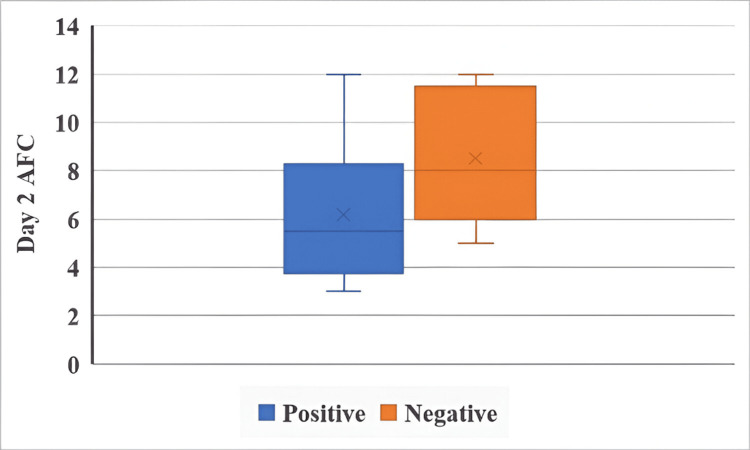
Day 2 AFC and IUI outcomes in the CC + HMG group The Mann-Whitney U test was used. p < 0.05 is considered statistically significant. AFC: antral follicle count, IUI: intrauterine insemination, CC: clomiphene citrate, HMG: human menopausal gonadotropin.

Day 2 AFC and IUI outcomes in both groups are illustrated in Figure 3 and Figure 4.

Day 2 AFC was not significantly associated with IUI outcome in the letrozole + HMG group (U = 96, p = 0.055), although the result approached significance. In contrast, a statistically significant association was observed in the CC + HMG group (U = 48, p = 0.037), indicating that day 2 AFC may influence IUI success in this group.

**Figure 5:**
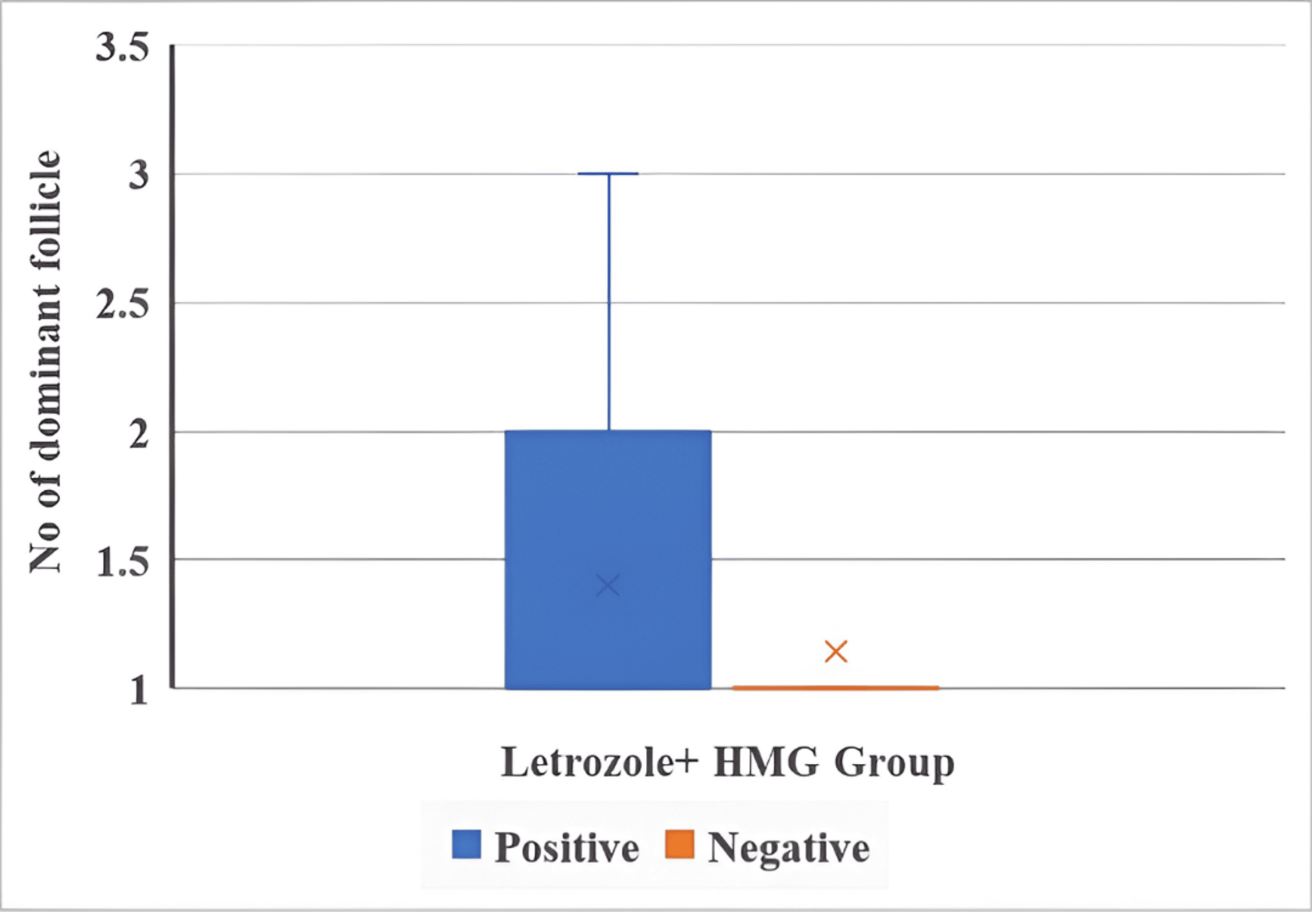
Number of dominant follicles and IUI outcomes in the letrozole + HMG group The Mann-Whitney U test was used. p < 0.05 is considered statistically significant. HMG: human menopausal gonadotropin.

**Figure 6:**
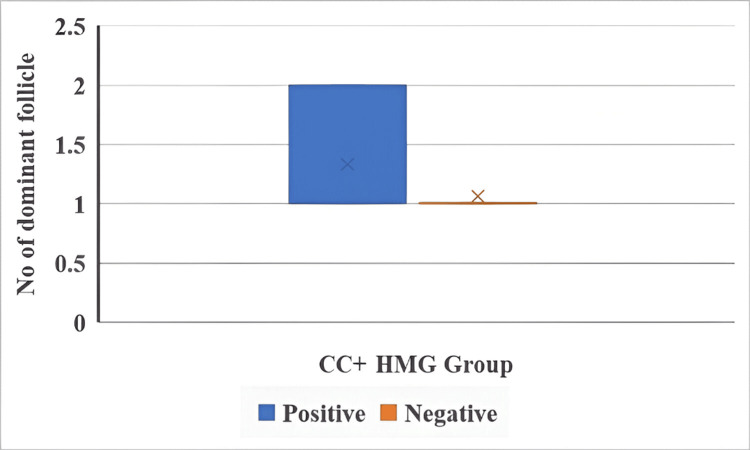
Number of dominant follicles and IUI outcomes in the CC + HMG group The Mann-Whitney U test was used. p < 0.05 is considered statistically significant. IUI: intrauterine insemination, CC: clomiphene citrate, HMG: human menopausal gonadotropin.

The number of dominant follicles and IUI outcomes in both groups are illustrated in Figure 5 and Figure 6.

The number of dominant follicles was not significantly associated with IUI outcomes in either the letrozole + HMG group (U = 114, p = 0.182) or the CC + HMG group (U = 122, p = 0.273).

## Discussion

The present study was designed to observe the effect of minimal stimulation using HMG with CC versus letrozole for ovulation induction in CC-resistant anovulatory infertility. There was no significant difference in IUI outcomes between the two groups, suggesting that both the letrozole + HMG and CC + HMG combinations are equally effective in achieving pregnancy.

There was no significant difference in the basic demographic characteristics between the groups. Similarly, no significant differences were found in mean follicular diameter, number of dominant follicles, or endometrial thickness, indicating that both treatment protocols are comparably effective in achieving optimal endometrial conditions for implantation. However, another study reported a significant difference in endometrial thickness between the two groups [14]. Additionally, in Group B, two patients developed more than two dominant follicles, which may be attributed to increased gonadotropin secretion stimulated by CC, potentially leading to the development of multiple follicles.

Two studies conducted on larger populations using the same drug combinations also reported pregnancy outcomes comparable to those in our study, although the baseline characteristics of patients and the duration of infertility differed between the groups in those studies [4,15]. However, another study found a statistically significant difference in pregnancy rates between the two groups. The variation in IUI outcomes compared to our findings might be attributed to their larger sample size and shorter mean duration of infertility (2.5 years) [15].

Our study found that the number of dominant follicles was not significantly associated with IUI outcomes in either Group A or Group B. Day 2 AFC was also not significantly associated with IUI outcomes in Group A; however, a significant association was observed in Group B. This suggests that pregnancy may still be achieved in women with low AFC through controlled stimulation using a CC and gonadotropin combination regimen. A study by Mathes et al. similarly found that antral follicle count was significantly associated with IUI outcomes [16]. In our study, the duration of infertility was inversely associated with IUI success in Group A, while no such association was observed in Group B. This may indicate that IUI success rates could decline as the duration of infertility increases. Supporting this, a study conducted in China also reported that infertility lasting more than five years was associated with a significantly lower pregnancy rate compared to shorter durations (95% CI: 0.817-0.991, p = 0.043). Additionally, that study found pregnancy rates were notably lower in cycles with a single pre-ovulatory follicle compared to those with multiple follicles [17].

In this study, no significant difference was observed in IUI outcomes between the two groups, suggesting that both combinations are equally effective in achieving pregnancy. Thakur and Pradhan [15], in a study conducted on a larger population of 180 infertile women in Iran, similarly found that letrozole could induce ovulation comparably to CC without adverse effects on the endometrium, and with a comparable pregnancy rate, consistent with the findings of our study. However, Pourali et al. [13] reported a statistically significant difference in pregnancy rates between the two groups (p = 0.022). This variation in outcome compared to our findings could be attributed to the larger sample size and shorter mean duration of infertility (2.5 years) in their study. It is known that CC, by increasing estrogen levels during ovarian stimulation, may have a detrimental effect on fertility treatment outcomes. Its anti-estrogenic action on the endometrium can lead to reduced endometrial receptivity and an increased risk of early pregnancy loss due to ER depletion. On the other hand, aromatase inhibitors like letrozole reduce estrogen synthesis, potentially mitigating these negative effects and improving follicular responsiveness to FSH. Differences in endometrial thickness and treatment outcomes may also stem from underlying ovulatory dysfunction. Nonetheless, combining either CC or letrozole with HMG appears to offset these limitations. Both our study and that by Thakur and Pradhan [15] support the conclusion that CC + HMG and letrozole + HMG regimens are equally effective in inducing ovulation in women with CC-resistant PCOS.

Limitation of the study

The study could have been strengthened by a larger sample size; however, this was limited by time constraints, as it was conducted as part of a postgraduate dissertation with a fixed study duration.

## Conclusions

To conclude, both letrozole and CC in combination with HMG are equally effective in inducing ovulation by promoting the development of ovarian follicles of optimal size and number and achieving adequate endometrial thickness for implantation. Thus, both regimens are equally effective in achieving successful IUI outcomes in women with PCOS and CC-resistant anovulatory infertility. The study also suggests that IUI success decreases with increasing duration of infertility and that pregnancy can still be achieved in women with low AFC through controlled ovarian stimulation using gonadotropin-based combination regimens.
